# Discharge Mobility After Osteoporotic Thoracolumbar Fractures Using a 2-Stage Machine Learning Prediction Model: Prospective Cohort Study

**DOI:** 10.2196/100486

**Published:** 2026-10-29

**Authors:** Anna Voelker, Dina Wilma Wiersbicki, Christoph-Eckhard Heyde, Tim Stelzner, Erik Schreiber, Thomas Neumuth, Juliane Neumann

**Affiliations:** 1 Department for Orthopedics, Trauma and Plastic Surgery, University Hospital Leipzig, Liebigstrasse 20, Leipzig, Saxony, 04103, Germany, 49 03419723000, 49 03419723009; 2 Innovation Center Computer Assisted Surgery (ICCAS), Medizinische Fakultät, Leipzig University, Leipzig, Saxony, Germany

**Keywords:** osteoporotic fractures, vertebral fractures, patient discharge, mobility limitation, machine learning, risk assessment, geriatric assessment, clinical prediction rules, functional outcome, spine

## Abstract

**Background:**

Discharge mobility after osteoporotic vertebral fractures is heterogeneous and difficult to predict at admission. Machine learning offers structured approaches for multiclass functional outcome prediction, but existing models predominantly address binary end points and report global performance metrics without explicit analysis of the error structure.

**Objective:**

This study aimed to develop and evaluate a 2-stage machine learning framework with a predefined decision policy designed to reduce extreme (LOW-HIGH) misclassification for graded discharge mobility prediction in hospitalized patients with osteoporotic thoracolumbar fractures.

**Methods:**

This prospective, single-center cohort study enrolled consecutive patients aged 65 years or older admitted for acute osteoporotic thoracic or lumbar fractures (N=158). Discharge mobility was categorized as LOW (<50 m or nonambulatory), MID (50‐500 m), or HIGH (>500 m or stair climbing). Clinical associations were analyzed using proportional odds ordinal regression. A predefined 2-stage architecture combined gradient-boosted trees (stage A: HIGH vs non-HIGH) and a random forest classifier (stage L: LOW vs non-LOW). The final 3-class prediction was assigned by a predefined decision policy designed to reduce clinically relevant extreme (LOW-HIGH) misclassification. All preprocessing steps, including fold-wise multiple imputation, were performed within training partitions only. The decision policy was additionally reevaluated in a nested cross-validation with fold-internal threshold selection across 10 repeated partitions.

**Results:**

Prefracture HIGH mobility was the only significant independent predictor of discharge mobility (odds ratio [OR] 3.38, 95% CI 1.68‐6.80; *P*<.001); age, comorbidity burden, fracture grade, and treatment were not. In the prespecified analysis with fixed thresholds, performance was moderate (accuracy 0.494; balanced accuracy 0.450; macro-*F*_1_ 0.444). Binary discrimination yielded an area under the curve of 0.693 (HIGH vs non-HIGH) and 0.625 (LOW vs non-LOW). Using the predefined decision policy, major LOW-HIGH errors were less frequent than with the prefracture mobility–only baseline (5 vs 22), and no HIGH-to-LOW misclassification occurred in the prespecified analysis. In the leakage-free nested cross-validation, macro-*F*_1_ was 0.388 (SD 0.031) and major errors had a mean of 8.3 (SD 3.4) vs 22.5 (SD 1.1) for the baseline, with fewer major errors in all 10 partitions. HIGH-to-LOW errors had a mean of 2.6 (SD 2.2) in this analysis.

**Conclusions:**

Discharge mobility after osteoporotic thoracolumbar fractures is primarily determined by prefracture functional reserve rather than fracture severity or comorbidity. A 2-stage machine learning framework achieved moderate multiclass discrimination and reduced extreme LOW-HIGH misclassification relative to a mobility-only baseline; this reduction persisted under nested cross-validation, whereas the absence of HIGH-to-LOW errors was specific to the prespecified partition. External validation is required before clinical deployment.

## Introduction

### Background

Osteoporotic vertebral fractures (OVFs) represent one of the most common fragility fractures in older adults and are associated with substantial morbidity, functional decline, and loss of independence. The affected population is typically characterized by advanced age, a female predominance, and multimorbidity, reflecting the epidemiology of vertebral fragility fractures in aging societies [1,2]. Beyond acute pain, OVFs frequently result in impaired mobility and reduced activities of daily living, with a considerable impact on discharge disposition and short-term recovery. Reliable prediction of discharge mobility from admission data would enable earlier targeting of rehabilitation resources, yet functional trajectories remain difficult to capture: individual clinical predictors show only a modest predictive signal, and recovery differs substantially between patients with ostensibly similar admission profiles.

Functional trajectories after hospitalization for OVF are heterogeneous. While fracture severity and treatment strategy guide therapeutic decision-making, growing evidence suggests that baseline mobility and frailty-related factors substantially influence postfracture functional outcomes [3-5]. However, functional reserve does not divide neatly into 2 states—gradations between mobility levels carry distinct implications for discharge planning, and modeling these distinctions as a multiclass outcome raises questions about class imbalance, threshold selection, and which prediction errors matter most clinically.

### Prior Work

Prefracture mobility, comorbidity burden, and frailty have been associated with postfracture functional status [6]. Frailty in particular has been linked to increased disability and reduced health-related quality of life in patients with vertebral compression fractures. Nutritional status, rehabilitation timing, and mobilization strategies may further influence discharge outcomes in older fracture populations [7,8].

Machine learning models have been applied to predict discharge outcomes in spine surgery and fracture populations using routine clinical variables [9,10], including functional impairment at discharge in OVF [4], and comparable approaches have predicted residual symptoms following OVF treatment [11]. These models share a common design: a binary end point derived by dichotomizing functional scales, evaluated by aggregate metrics such as the area under the curve (AUC) [12]. This framing obscures a clinically relevant distinction—confusing a patient with high mobility with one who is nearly nonambulatory is a qualitatively different error than misclassifying adjacent mobility categories, yet existing models do not differentiate between these outcomes. Using the same data for both threshold selection and performance estimation is a recognized form of data leakage that leads to overoptimistic results [13,14], and, to our knowledge, no published OVF prediction model has explicitly incorporated a predefined decision policy for reducing clinically relevant high-consequence prediction errors.

### Study Objectives

The present study therefore modeled discharge mobility as a 3-level ordinal outcome (LOW, MID, and HIGH) and developed a 2-stage machine learning architecture in which a predefined decision policy governs the final 3-class assignment while reducing clinically relevant extreme misclassifications. Ordinal regression was used in parallel for clinical characterization of predictor associations. The primary methodological aim was to demonstrate that explicit error-type control is achievable in a real-world multiclass clinical prediction setting without sacrificing interpretability.

## Methods

### Study Design and Patient Population

This prospective, single-center cohort study was conducted at a university hospital between June 2023 and December 2024. Consecutive patients aged 65 years or older who required inpatient treatment for an acute symptomatic thoracic or lumbar OVF were eligible. An osteoporotic fracture (OF) was defined as a fracture resulting from low-energy trauma or occurring without an adequate trauma mechanism.

Exclusion criteria comprised high-energy trauma, pathological fractures, spinal infections, or incomplete datasets regarding the primary outcome or core predictors. Treatment was performed according to routine clinical decision-making by the treating team [15,16].

### Ethical Considerations

The study protocol was approved by the institutional ethics committee from the Faculty of Medicine, Leipzig University (approval 214/23-ek), and the study was conducted in accordance with the Declaration of Helsinki. Written informed consent was obtained from all participants prior to enrollment.

### Outcome Definition

The primary outcome was functional mobility at hospital discharge, categorized into 3 clinically meaningful groups: LOW (walking distance <50 m, transfer only, or bedridden), MID (walking distance 50‐500 m), and HIGH (walking distance >500 m or ability to climb stairs). These category boundaries were defined a priori by clinical consensus and were not derived from the study data. They were based on functionally meaningful walking distances described in previous mobility and rehabilitation research (approximately 50 m and 500 m, with stair climbing indicating higher functional mobility) [17,18]. No validated discharge-mobility classification specific to this population has been established. This 3-level ordinal classification served as the outcome variable for all analyses. Discharge mobility was assessed by the treating physiotherapists through direct observation and was routinely documented in standardized physiotherapy progress notes as part of clinical care.

### Predictor Variables and Feature Specification

Only variables available at admission or within −2 to +3 days relative to hospital admission were considered. Postoperative or in-hospital course variables were excluded.

1. Constitution characteristics: age, sex, BMI, and Charlson Comorbidity Index (CCI).
2. Baseline functional status: prefracture mobility was recorded on a 6-level ordinal scale (1=bedridden to 6=walking >500 m or stair climbing). For machine learning, mobility was included as the original ordinal variable (1-6) and as 3 binary indicators (LOW, MID, and HIGH). For regression analyses, it was dichotomized (HIGH vs non-HIGH).
3. Fracture characteristics and treatment: OF grade per 1-grade increment [19], neurological deficit at admission, and treatment modality (operative vs conservative). Prespecified interaction terms (prefracture mobility × CCI, neurological deficit × prefracture mobility, treatment × OF grade) were defined a priori.
4. Laboratory parameters: grouped into domains reflecting inflammation, hematologic status, metabolic/electrolyte balance, renal and hepatic function, and coagulation. The first available value within −2 to +3 days from admission was used (except 25-hydroxyvitamin D, accepted between admission and discharge). Duplicate assay sources were consolidated into single variables. Hemoglobin is reported in mmol/L (1 mmol/L corresponds to approximately 1.61 g/dL).

Feature inclusion was determined a priori based on clinical reasoning and predefined availability criteria. The final feature set was fixed before model training and stored as a frozen feature matrix. No data-driven variable selection procedures were applied.

### Descriptive Statistics

Continuous variables are reported as median with IQR. Categorical variables are presented as counts and percentages. Comparisons across discharge mobility categories (LOW, MID, and HIGH) were performed using Kruskal-Wallis tests for continuous variables and *χ*^2^ tests or Fisher exact tests for categorical variables, as appropriate.

### Statistical Analysis

Regression analyses were conducted post hoc for clinical characterization and were independent of machine learning model development. Associations between selected clinical variables (age, CCI, OF grade, treatment modality, and dichotomized prefracture mobility) and discharge mobility were analyzed using proportional odds ordinal regression (OrderedModel). The proportional odds assumption was formally assessed prior to final model specification.

Additional binary logistic regression models (HIGH vs non-HIGH and LOW vs non-LOW) were estimated as supplementary analyses. Complete-case analysis was applied; as the regression model variables had no missing values, no cases were excluded.

### Machine Learning Model Development

A predefined 2-stage machine learning architecture was used to predict discharge mobility (Figure 1).

Stage A estimated the probability of HIGH vs non-HIGH mobility using a gradient-boosted decision tree model (HistGradientBoostingClassifier). Stage L estimated the probability of LOW vs non-LOW mobility using a random forest classifier (RandomForestClassifier), selected for its robustness to class imbalance and heterogeneous predictor effects. Missing values were imputed within the training folds of each cross-validation split, and the fitted imputers were applied to the held-out fold. Stage A used median imputation for continuous variables and most-frequent imputation with one-hot encoding for categorical variables; stage L used multivariable iterative imputation (multiple imputation by chained equations).

Structural hyperparameters for both stages were determined during a preceding model-development phase and were then applied unchanged across all 5 cross-validation folds. Stage A used fixed gradient-boosting hyperparameters without class weighting, and stage L used fixed random-forest hyperparameters with balanced class weights. No oversampling or undersampling procedures were applied to address class imbalance. No additional L1/L2 penalization was applied.

**Figure 1.**
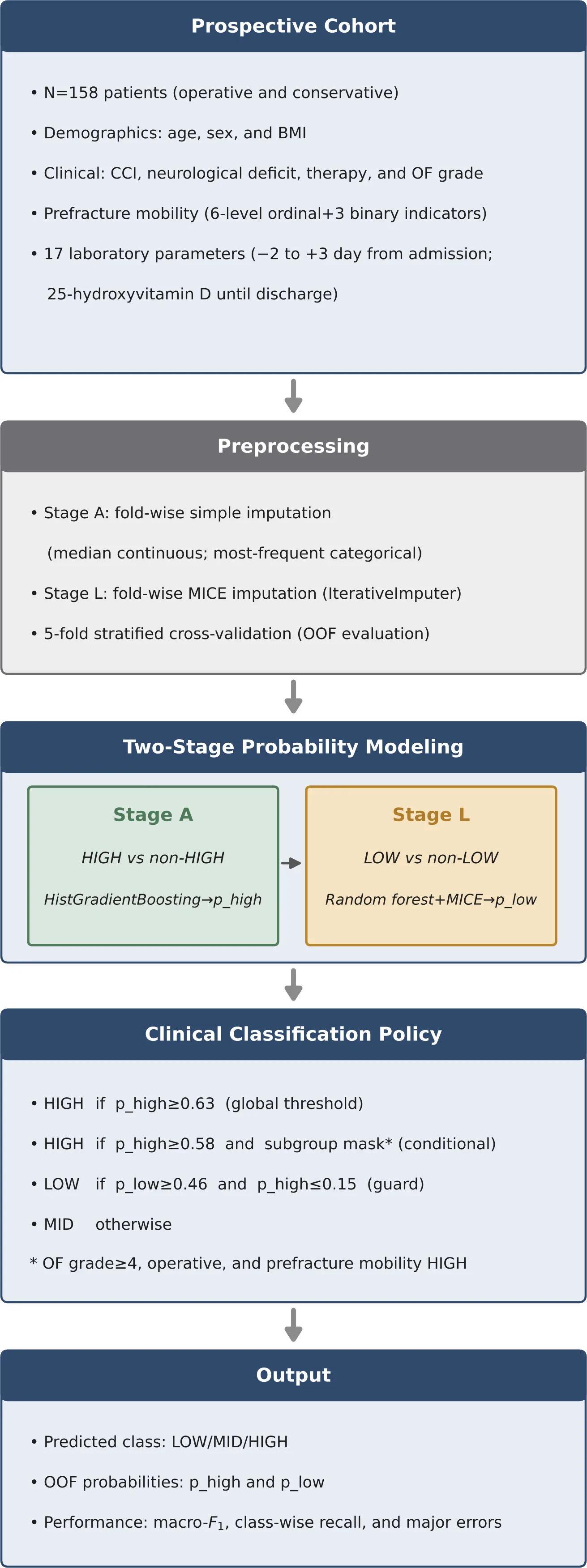
Study workflow and 2-stage risk stratification framework for discharge mobility prediction. The model estimates probabilities for HIGH and LOW mobility and derives the final 3-class prediction via a predefined decision policy. CCI: Charlson Comorbidity Index; MICE: multivariable iterative imputation; OF: osteoporotic fracture; OOF: out-of-fold.

### Threshold Selection and Policy Optimization

The 2 models output only class probabilities (*P*_high_ and *P*_low_); the final 3-class label is assigned by a predefined decision policy.

Final 3-class predictions were derived from the 2 probability estimates (*P*_high_ and *P*_low_). HIGH was assigned for *P*_high_ values greater than or equal to 0.63 or *P*_high_ values greater than or equal to 0.58 in patients with OF grade 4 or higher, operative treatment, and high prefracture mobility. LOW was assigned only for *P*_low_ values greater than or equal to 0.46 and *P*_high_ values less than or equal to 0.15; all remaining cases were assigned MID.

The decision-policy thresholds were determined during model development from the pooled out-of-fold predictions. Candidate configurations were restricted to those with a HIGH recall of at least 0.40, a LOW recall of at least 0.18, and a predicted MID proportion of at most 0.75. Among the remaining configurations, the one with the fewest major errors (LOW-HIGH) was selected, with macro-*F*_1_, HIGH recall, and LOW recall as successive tie-breakers [20]. If no configuration met these constraints, the same ranking was applied without them. The absence of HIGH-to-LOW errors was not imposed as a constraint. The resulting thresholds were fixed and were not reestimated within each fold. The policy structure and this selection procedure were specified in advance, whereas the numerical thresholds were derived from the data as described.

### Model Evaluation

Model performance was estimated from 5-fold stratified cross-validation using the out-of-fold predictions. Imputation and model fitting were performed within the training folds; the structural hyperparameters and decision-policy thresholds had been fixed during the preceding model-development phase. Major errors were defined a priori as misclassification between LOW and HIGH. Secondary metrics included class-wise recall and macro-*F*_1_. All 31 candidate predictors were specified a priori on clinical grounds. The number of events was 70 (HIGH), 59 (MID), and 29 (LOW), corresponding to 2.26 and 0.94 events per candidate predictor for the HIGH and LOW stages, respectively [21].

To assess the added value of the 2-stage framework, we specified a baseline multinomial logistic regression model using the 3-level prefracture mobility category as the sole predictor (MID as the reference), with balanced class weights and L2 regularization (C=1.0), evaluated using 5-fold stratified cross-validation. Baseline predictions were assigned based on the maximum predicted probability; no threshold-based decision policy equivalent to that of the 2-stage framework was applied.

Calibration of both stages was assessed using the logistic calibration slope and intercept with 95% CIs, based on out-of-fold predictions, and displayed as calibration plots [22]. No post hoc probability recalibration was applied. Analyses were conducted in Python 3.12.7 using scikit-learn 1.8.0, pandas 3.0.0, NumPy 2.4.1, and Matplotlib 3.10.8, with fixed random seeds for reproducibility.

### Nested Evaluation of the Decision Policy

Because the thresholds were selected on the same out-of-fold predictions used for reporting, the decision policy was additionally evaluated in a nested design. Within each fold of an outer 5-fold cross-validation, stratified by the 3-level outcome so that both stages shared the same partition, both stages were refitted in an inner 5-fold cross-validation of the training partition. Thresholds were then selected from the inner out-of-fold predictions using the procedure described above and applied to the held-out outer fold. The entire procedure was repeated across ten 5-fold cross-validation resampling partitions, each covering all 158 patients. On the same partitions, the fixed thresholds of the final policy and the baseline model were evaluated for comparison. Structural hyperparameters remained fixed throughout and were not selected within the nested loops.

### Feature Importance

Feature importance was assessed for both stages using permutation importance computed on the held-out test fold of each cross-validation split and summarized across folds (30 repeats, receiver operating characteristic–AUC scoring; median with IQR). Permutation importance was preferred over impurity-based measures, which are biased toward continuous and high-cardinality variables. Importance measures were used for interpretation only and did not influence model specification, feature selection, or threshold determination.

## Results

### Patient Cohort

The study included 158 patients aged 65 years or older with acute osteoporotic thoracolumbar fractures (Table 1). Overall missingness was low (44/4898, 0.9% feature cells) and confined to 6 laboratory variables, with vitamin D showing the highest variable-specific proportion of missing values (29/158, 18.4%). The proportion of operatively treated patients increased from 0% (0/5) in OF grade 1 to 96.2% (25/26) in OF grade 5 (*P*<.001).

**Table 1.** Baseline characteristics stratified by discharge mobility.^a^

Variable	Overall (N=158)	LOW (n=29)	MID (n=59)	HIGH (n=70)	*P* value
Age (y), median (IQR)	82.0 (76.1‐85.8)	82.9 (77.1‐88.0)	83.2 (80.2‐86.3)	80.4 (74.1‐84.5)	.02
Sex (female), n (%)	96 (60.8)	17 (58.6)	44 (74.6)	35 (50)	.02
BMI (kg/m²), median (IQR)	24.9 (22.5‐28.0)	24.0 (22.2-28.7)	26.0 (22.9-29.1)	24.8 (22.6-27.2)	.43
Charlson Comorbidity Index, median (IQR)	7 (5-10)	8 (6-11)	8 (6-10)	7 (5-8)	.01
Neurological deficit, n (%)	17 (10.8)	5 (17.2)	7 (11.9)	5 (7.1)	.27
Prefracture mobility LOW, n (%)	32 (20.3)	11 (37.9)	16 (27.1)	5 (7.1)	N/A^b^
Prefracture mobility MID, n (%)	13 (8.2)	3 (10.3)	6 (10.2)	4 (5.7)	<.001^c^
Prefracture mobility HIGH, n (%)	113 (71.5)	15 (51.7)	37 (62.7)	61 (87.1)	N/A
CRP (mg/L), median (IQR)	6.0 (2.3‐32.8)	7.2 (2.6‐37.5)	14.5 (3.4‐38.2)	4.5 (1.8‐16.3)	.04
Hemoglobin (mmol/L), median (IQR)	7.9 (6.9‐8.5)	7.2 (6.2‐7.9)	7.8 (6.7‐8.3)	8.3 (7.6‐8.8)	<.001
eGFR (mL/min/1.73 m²), median (IQR)	65.0 (48.2‐80.0)	58.0 (43.0–80.0)	56.0 (39.5–74.0)	70.5 (61.2–83.8)	<.001
OF^d^, n (%)					
Grade 1	5 (3.2)	—^e^	—	—	
Grade 2	24 (15.2)	—	—	—	
Grade 3	45 (28.5)	—	—	—	
Grade 4	58 (36.7)	—	—	—	
Grade 5	26 (16.5)	—	—	—	
Operative treatment, n (%)	80 (50.6)	13 (44.8)	26 (44.1)	41 (58.6)	.21

a Laboratory variables were limited a priori to 3 clinically interpretable domains to avoid redundancy: systemic inflammation (C-reactive protein [CRP]), anemia (hemoglobin), and renal function (estimated glomerular filtration rate [eGFR])*.*

b Not applicable.

c *P* value for the overall comparison of prefracture mobility categories.

d OF: osteoporotic fracture.

e Not available.

### Multivariable Ordinal Regression

Prefracture HIGH mobility was independently associated with higher discharge mobility (odds ratio [OR] 3.38, 95% CI 1.68-6.80; *P*<.001). In contrast, age, CCI, OF grade, and operative treatment were not significantly associated with discharge mobility (age per 10 y; OR 0.855, 95% CI 0.512-1.430; CCI OR 0.896, 95% CI 0.787-1.021 per point; OF grade OR 0.921, 95% CI 0.649-1.308; treatment OR 1.995, 95% CI 0.953-4.174).

Overall model fit was modest (Akaike information criterion [AIC] 317.2; McFadden R² 0.077), and the proportional odds assumption was met (Brant test *P*=.81; Figure 2). A null model restricted to prefracture mobility alone yielded McFadden *R*² 0.049 and AIC 318.5, indicating only marginal incremental fit from additional clinical predictors (ΔAIC=1.3).

**Figure 2.**
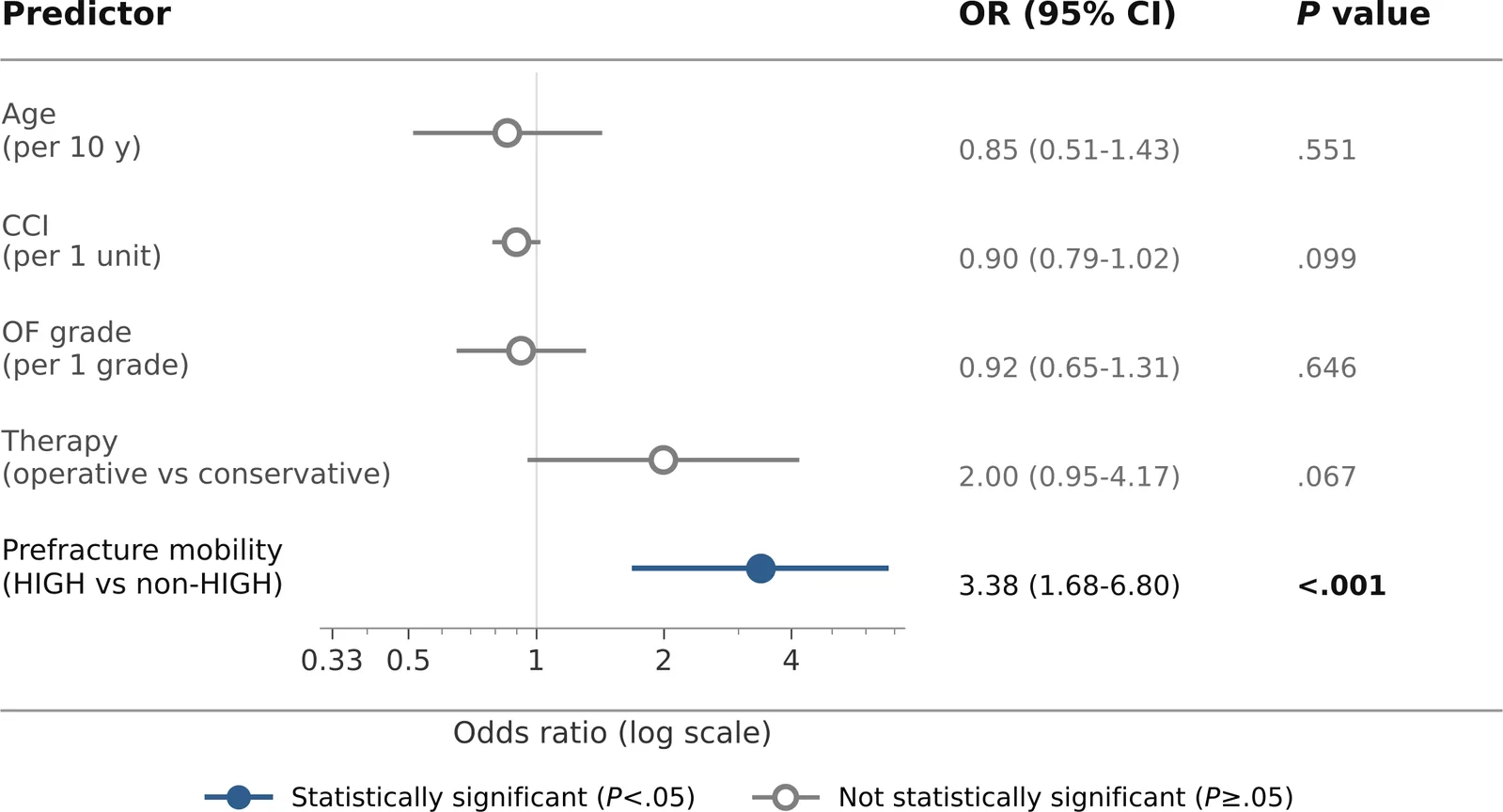
Multivariable ordinal logistic regression analysis of discharge mobility. Forest plot of the proportional odds model (LOW<MID<HIGH). Prefracture mobility (HIGH vs non-HIGH) was independently associated with higher discharge mobility (odds ratio [OR] 3.38, 95% CI 1.68-6.80; *P*<.001). Age, Charlson Comorbidity Index (CCI), osteoporotic fracture (OF) grade, and treatment modality were not statistically significant. ORs are displayed on a logarithmic scale; the vertical line indicates no effect (OR=1).

### Machine Learning Model Performance

Overall accuracy of the 2-stage model was 0.494, with balanced accuracy of 0.450 and macro-*F*_1_ of 0.444. Detailed performance, discrimination, and calibration metrics are summarized in Table 2, and calibration plots are shown in Figure 3. Calibration slopes were below 1 for both stages, indicating imperfect calibration of the predicted probabilities. These values refer to the prespecified analysis using fixed thresholds; leakage-free estimates for the complete threshold-selection procedure are reported below.

**Table 2.** Performance of the 2-stage prediction model.^a^

Metric	Values
Accuracy	0.494
Balanced accuracy	0.450
Macro-*F*_1_	0.444
Recall	
LOW	0.207
MID	0.729
HIGH	0.414
Precision	
LOW	0.462
MID	0.422
HIGH	0.674
AUC^b^	
HIGH	0.693
LOW	0.625
Brier score	
HIGH	0.227
LOW	0.181
Calibration slope (95% CI)	
HIGH	0.590 (0.330 to 0.909)
LOW	0.831 (0.156 to 1.600)
Calibration intercept (95% CI)	
HIGH	−0.044 (−0.169 to 0.115)
LOW	−1.059 (−1.407 to −0.741)

a Overall, class-wise, discrimination, and calibration metrics obtained from 5-fold cross-validation*.*

b AUC: area under the curve.

**Figure 3.**
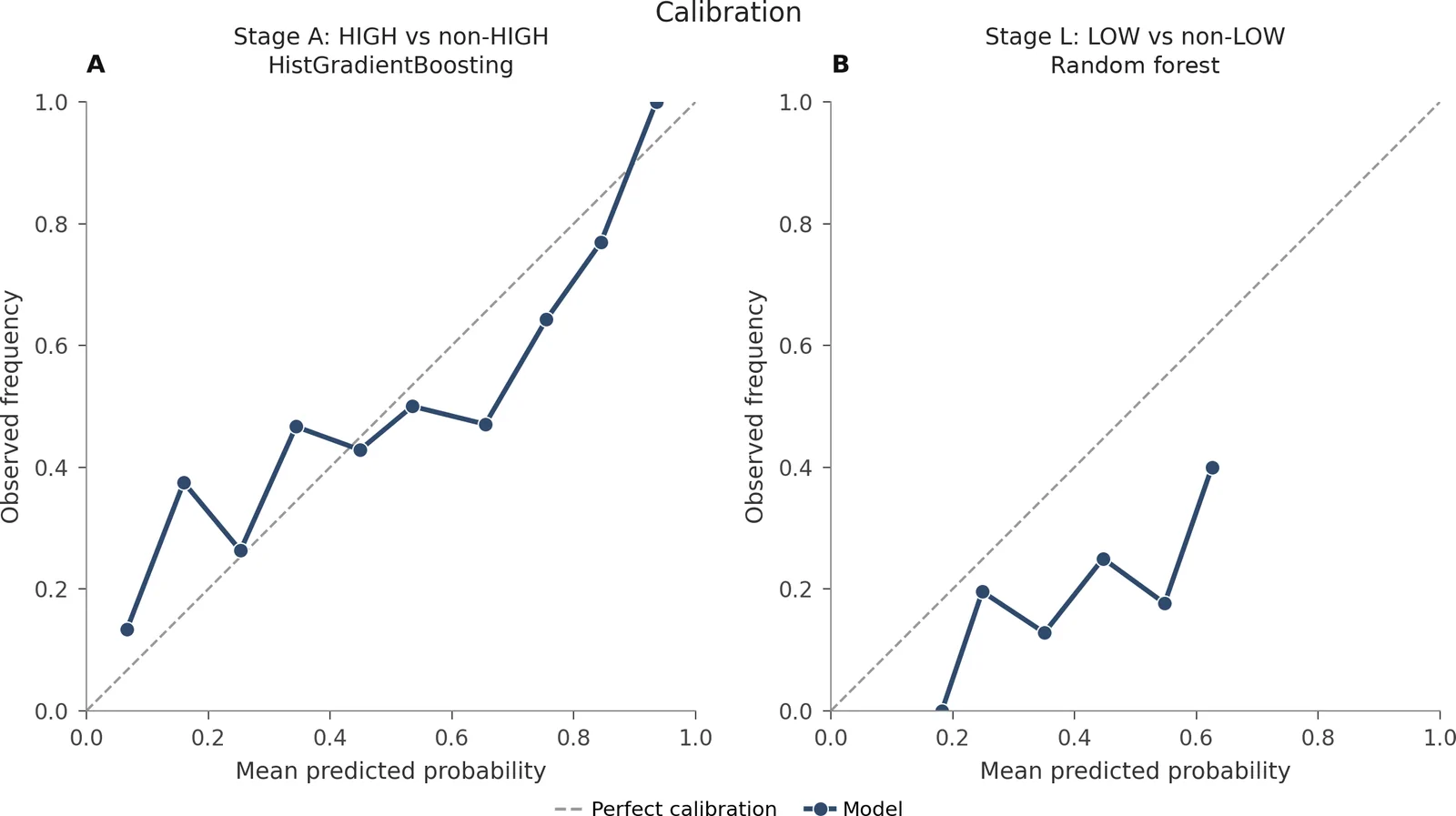
Calibration plots for stage A (HIGH vs non-HIGH) and stage L (LOW vs non-LOW) based on cross-validated out-of-fold predictions. Predicted probabilities are plotted against observed frequencies, with the diagonal indicating perfect calibration. The logistic calibration slope and intercept were 0.590 and −0.044 for stage A (Brier score 0.227) and 0.831 and −1.059 for stage L (Brier score 0.181).

Compared with the prefracture mobility–only baseline, the 2-stage framework showed higher macro-*F*_1_ (0.444 vs 0.349) but only modest differences in discrimination, whereas calibration slopes were closer to 1 for the baseline (Table 3). MID recall was 0.73 vs 0.02 for the baseline, and major LOW-HIGH errors were 5 vs 22, including HIGH-to-LOW errors of 0 vs 7.

**Table 3.** Comparison of the baseline (prefracture mobility only) and 2-stage models.^a^

Metric	Baseline (prefracture mobility)	Two-stage model
Macro-*F*_1_ (95% CI)	0.349 (0.290‐0.407)	0.444 (0.355‐0.526)
AUC^b^		
HIGH vs non-HIGH (95% CI)	0.602 (0.510‐0.688)	0.693 (0.605‐0.776)
LOW vs non-LOW (95% CI)	0.586 (0.456‐0.714)	0.625 (0.519‐0.726)
Calibration slope		
HIGH (95% CI)	0.939 (0.426‐1.655)	0.590 (0.330‐0.909)
LOW (95% CI)	0.916 (0.107‐1.775)	0.831 (0.156‐1.600)
Major errors		
LOW-HIGH	22	5
HIGH→LOW	7	0
LOW→HIGH	15	5

a Values are point estimates with 95% bootstrap CIs, where shown*.*

b AUC: area under the curve.

### Confusion Matrix and Misclassification Patterns

LOW mobility was correctly identified in 6 of 29 (20.7%) cases, with most remaining cases classified as MID (18/29, 62.1%). MID mobility was correctly identified in 43 of 59 (72.9%) cases, and HIGH in 29 of 70 (41.4%) cases. No HIGH cases were misclassified as LOW (Figure 4).

**Figure 4.**
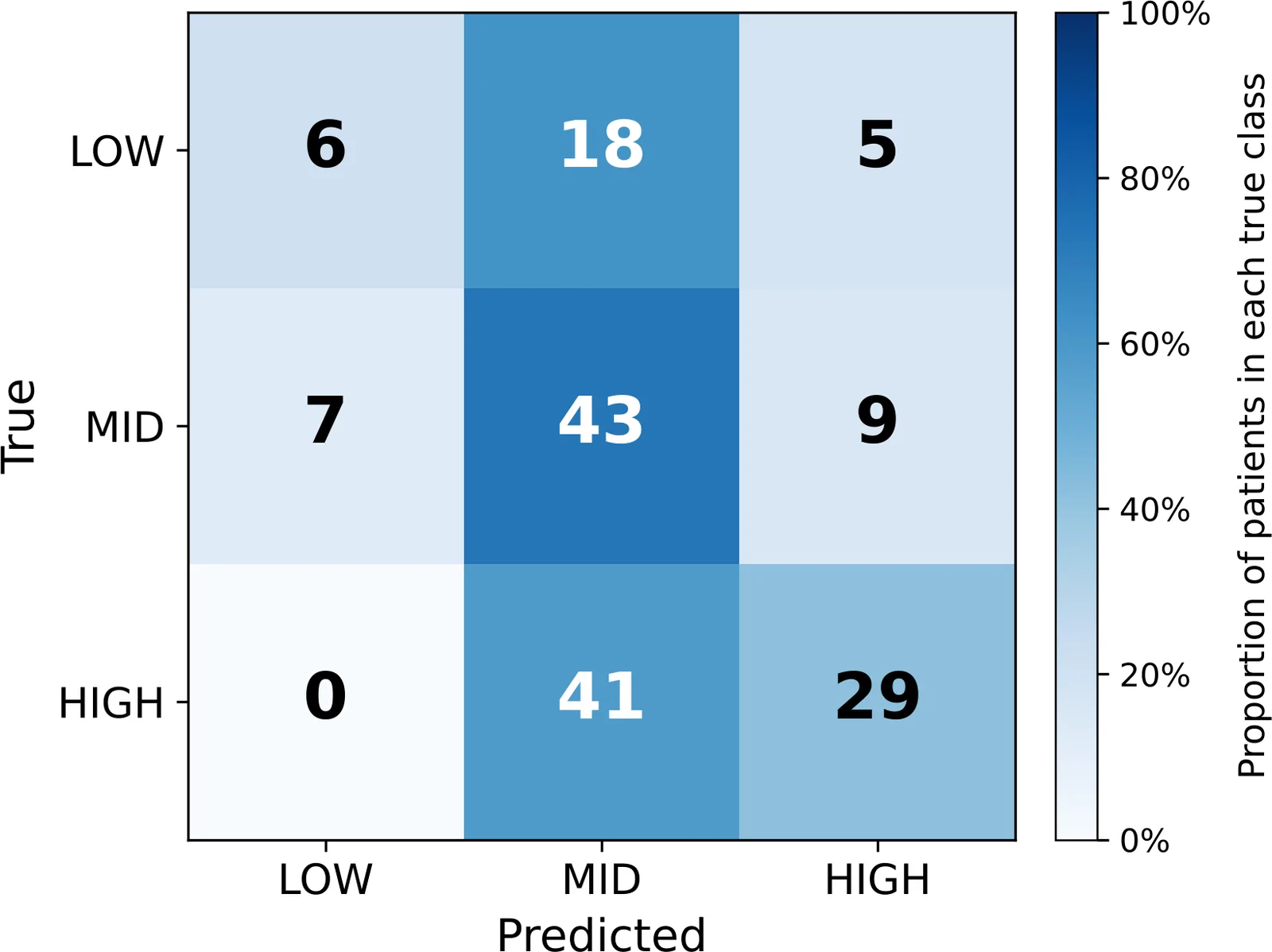
Confusion matrix of the 2-stage prediction model. Observed vs predicted discharge mobility categories (LOW, MID, and HIGH) based on cross-validated out-of-fold predictions. Cell values represent absolute patient counts.

### Clinical Strata Analysis

Observed and predicted discharge mobility distributions were examined across CCI strata, treatment categories, and predefined risk profiles. Across CCI strata, observed HIGH mobility decreased with increasing comorbidity (32/57, 56.1%; 29/61, 47.5%; and 9/40, 22.5% for CCI ≤6, 7‐9, and ≥10, respectively), whereas predicted HIGH proportions were consistently lower (26/57, 45.6%; 13/61, 21.3%; and 4/40, 10.0%). Similar patterns were observed across treatment groups and risk profiles, with predicted MID proportions exceeding observed MID proportions across all strata. Major errors (LOW-HIGH) remained limited across strata (CCI: 1/3/1; conservative: 1; operative: 4; high-risk: 1; low-risk: 0; Figure 5). Two predefined risk profiles were compared: a high-risk profile (high prefracture mobility, CCI ≥9, operative treatment; n=14) and a low-risk profile (high prefracture mobility, CCI≤6, conservative treatment; n=25).

**Figure 5.**
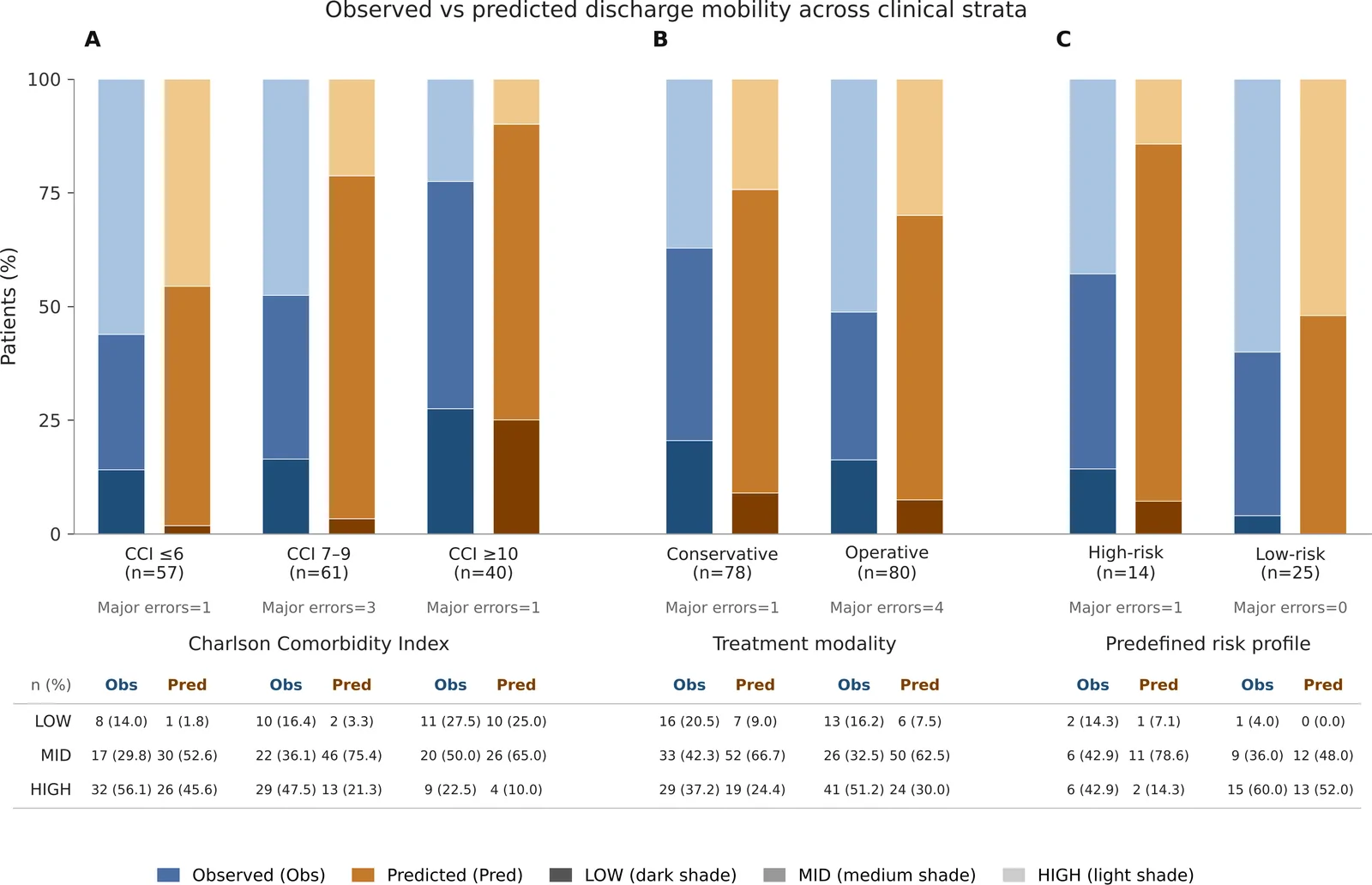
Observed and predicted discharge mobility across clinical strata. Distribution of LOW, MID, and HIGH discharge mobility across Charlson Comorbidity Index (CCI) strata, treatment modality, and predefined risk profiles, based on cross-validated out-of-fold predictions. Bars show percentages; the table below gives n (%). Major errors indicate LOW-HIGH misclassifications per stratum. High-risk profile: HIGH prefracture mobility, CCI ≥9, and operative treatment (n=14); low-risk profile: HIGH prefracture mobility, CCI ≤6, and conservative treatment (n=25).

### Detailed Analysis of LOW-to-HIGH Major Misclassifications

All 5 major misclassifications represented LOW-to-HIGH transitions. Predicted probabilities for HIGH mobility were consistently elevated (0.721-0.829), placing these cases within the upper 16% (26/158) of the cohort’s *P*_high_ distribution. The median *P*_high_ among misclassified patients (0.782, IQR 0.741-0.794) exceeded that of all true HIGH patients (0.516, IQR 0.325-0.728), and 74% (52/70) true HIGH cases had a lower predicted probability than the lowest misclassified case. No HIGH-to-LOW misclassifications occurred.

Clinically, 3 patients had high prefracture mobility and underwent operative treatment for OF grade 4 or higher fractures. Two showed markedly elevated inflammatory markers with reduced hemoglobin, and 1, managed conservatively, was of advanced age with a high comorbidity burden. No single structural factor alone explained the discrepancy between predicted and observed mobility (Table 4).

**Table 4.** Clinical and probability characteristics of LOW-to-HIGH major misclassifications.^a^

Age (y)	CCI^b^	Treatment	OF^c^ grade	Prefracture mobility (1-6)	CRP^d^ (mg/L)	Hemoglobin (mmol/L)	*p*_high_
80.5	7	Operative	4	3	1.3	9.1	0.829
84.1	7	Operative	5	6	46.6	5.4	0.741
81.9	9	Operative	5	6	6.8	8.6	0.794
79.8	4	Operative	5	6	82.6	6.2	0.721
94.5	11	Conservative	3	4	2.3	7.7	0.782

a Selected baseline clinical variables and corresponding *P*_high_ values for the 5 LOW-to-HIGH major errors.

b CCI: Charlson Comorbidity Index.

c OF: osteoporotic fracture.

d CRP: C-reactive protein.

### Clinical Characteristics of Adjacent Misclassifications

Among true HIGH patients misclassified as MID, patients were older and had a higher comorbidity burden, lower hemoglobin, reduced estimated glomerular filtration rate (eGFR), and lower *P*_high_ probabilities compared with correctly classified true HIGH patients (81.9 vs 76.6 y; CCI 8.0 vs 5.0; hemoglobin 7.70 vs 8.50 mmol/L; eGFR 64.0 vs 84.0 mL/min; *P*_high_ 0.361 vs 0.766). Among true MID patients misclassified as LOW, elevated C-reactive protein (CRP), lower hemoglobin, reduced renal function, and higher *P*_low_ probabilities were observed (CRP 52.9 vs 16.3 mg/L; hemoglobin 6.50 vs 7.80 mmol/L; eGFR 44.0 vs 55.0 mL/min; *P*_low_ 0.580 vs 0.382). No consistent pattern was observed for LOW-to-MID or MID-to-HIGH misclassifications (Figure 6).

**Figure 6.**
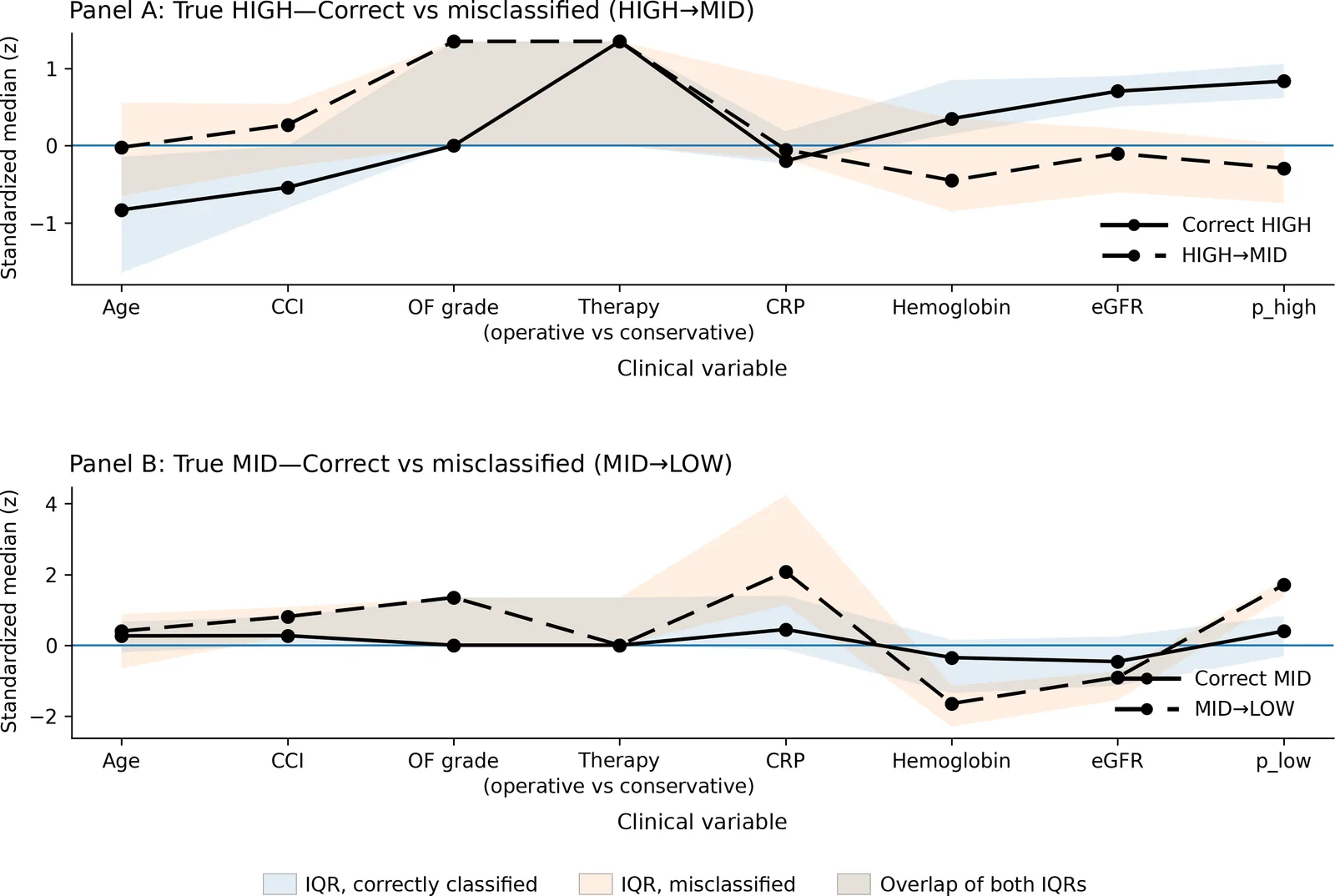
Clinical profiles of correctly classified and adjacent misclassified patients. Standardized median differences (*z* scores) of selected variables comparing correctly classified patients with adjacent misclassifications within the true HIGH (HIGH→MID) and true MID (MID→LOW) groups. Solid lines show the median of correctly classified patients, dashed lines show the median of misclassified patients, and shaded bands show the IQR of the respective group. Values are standardized (z) against the correctly classified group; for binary variables, this standardization is unstable. CCI: Charlson Comorbidity Index; CRP: C-reactive protein; eGFR: estimated glomerular filtration rate; OF: osteoporotic fracture.

### Variable Importance

In stage A (HIGH vs non-HIGH), test-fold permutation importance was highest for aspartate aminotransferase/glutamic-oxaloacetic transaminase and eGFR, followed by age and prefracture mobility. Comorbidity burden (CCI) and CRP contributed moderately, while the remaining variables showed low and increasingly uncertain importance across folds (Figure 7).

In stage L (LOW vs non-LOW), hematocrit showed the highest importance, followed by several prefracture mobility variables and the metabolic markers vitamin D and calcium. Beyond these highest-ranked features, importance was low and showed wide fold-to-fold variability, reflecting limited and heterogeneous predictive signal (Figure 7).

**Figure 7.**
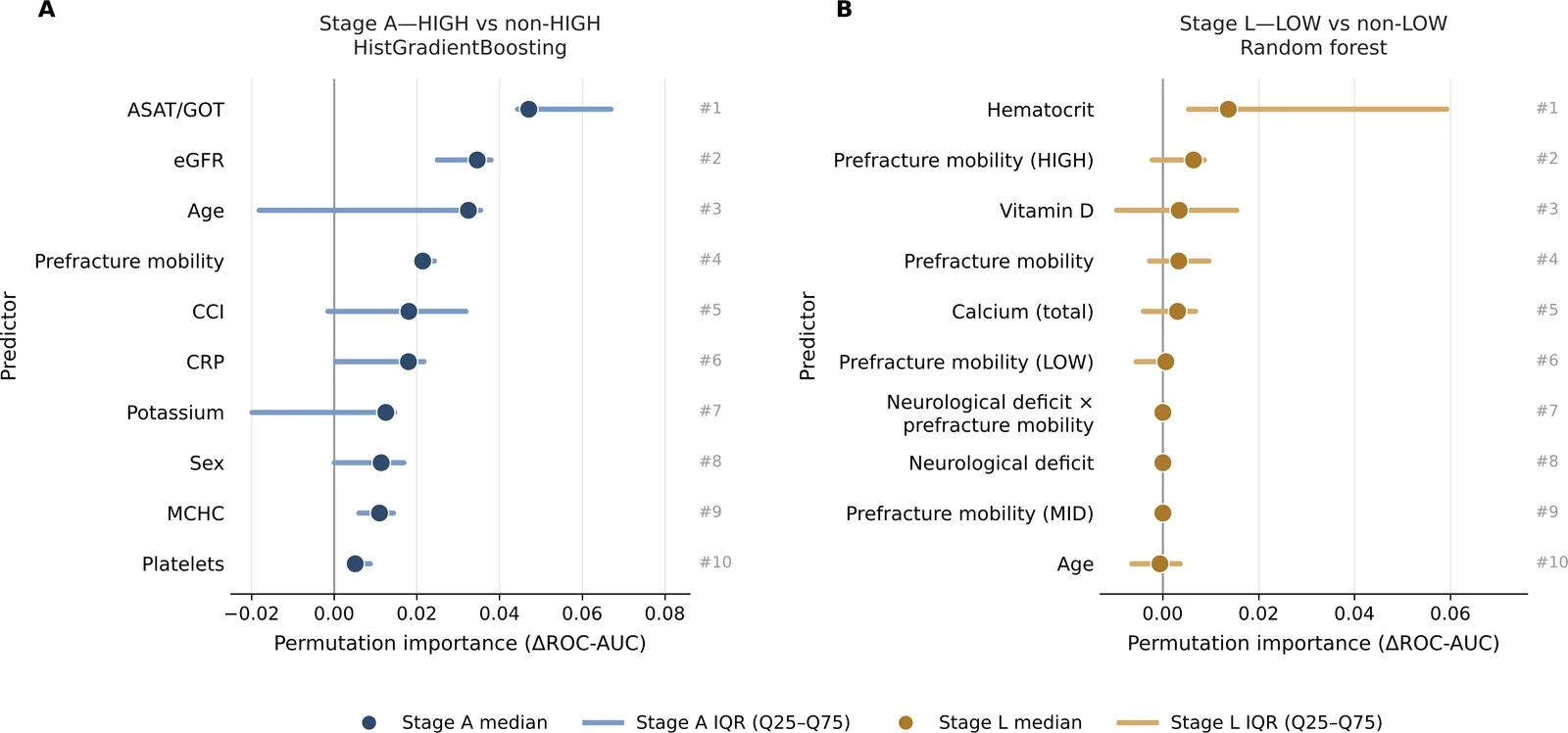
Permutation importance (change in receiver operating characteristic–area under the curve [ROC-AUC]) was computed on the held-out test fold of each cross-validation split (30 repeats) and is shown as the median (point) and IQR (Q25-Q75; line) across folds. The 10 highest-ranked predictors are displayed for each stage. Numbers on the right (#1-#10) indicate the importance rank of each predictor. ASAT/GOT: aspartate aminotransferase/glutamic-oxaloacetic transaminase; CCI: Charlson Comorbidity Index; CRP: C-reactive protein; eGFR: estimated glomerular filtration rate; MCHC: mean corpuscular hemoglobin concentration.

### Nested Evaluation of the Decision Policy

Across 10 partitions, nested threshold selection yielded a macro-*F*_1_ of 0.388 (SD 0.031), an accuracy of 0.457 (SD 0.038), and 8.3 (SD 3.4) major errors. These comprised 2.6 (SD 2.2) HIGH-to-LOW errors and 5.7 (SD 1.9) LOW-to-HIGH errors. No HIGH-to-LOW error occurred in 1 of 10 partitions.

Applying the fixed thresholds to the same partitions yielded a macro-*F*_1_ of 0.390 (SD 0.031) and 8.4 (SD 3.1) major errors. The baseline model yielded a macro-*F*_1_ of 0.350 (SD 0.009) and 22.5 (SD 1.1) major errors. The 2-stage framework produced fewer major errors in all 10 partitions (paired difference of −14.2, SD 3.1; Figure 8).

**Figure 8.**
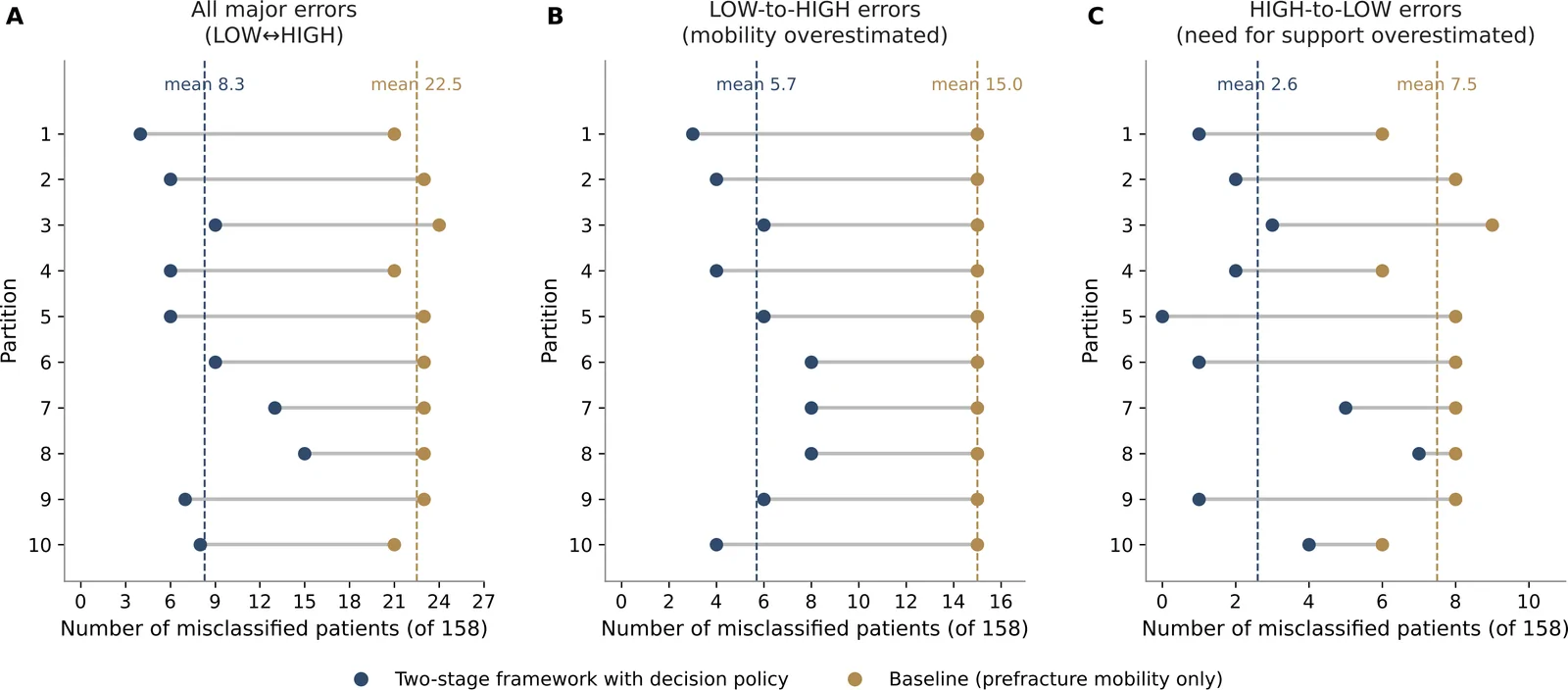
Misclassification between LOW and HIGH mobility across 10 repeated 5-fold cross-validation runs. Each row is one 5-fold cross-validation partition covering all 158 patients. Markers show the 2-stage framework, with thresholds selected within the training data of each outer fold, and the prefracture mobility–only baseline, evaluated on identical partitions and held-out patients; dashed lines mark the means across partitions. (A) All major errors (LOW-HIGH): 8.3 (SD 3.4) vs 22.5 (SD 1.1). (B) LOW-to-HIGH errors, in which mobility is overestimated: 5.7 (SD 1.9) vs 15.0 (SD 0.0). (C) HIGH-to-LOW errors, in which the need for support is overestimated: 2.6 (SD 2.2) vs 7.5 (SD 1.1). The panel scales differ.

Class-wise recall under nested evaluation was 0.117 for LOW (SD 0.037), 0.669 for MID (SD 0.068), and 0.419 for HIGH (SD 0.045). Thresholds selected within folds were close to the fixed values (with a median of 0.63 for *P*_high_ and 0.48 for *P*_low_). Results for each partition are provided in Multimedia Appendix 1.

## Discussion

### Principal Findings

Prefracture functional capacity was the dominant determinant of discharge mobility, irrespective of fracture grade and treatment strategy. Consistent with this, prefracture mobility was the only significant independent predictor in ordinal regression. In the machine-learning analyses, permutation importance ranked aspartate aminotransferase/glutamic-oxaloacetic transaminase and eGFR highest in stage A, followed by age and prefracture mobility, whereas hematocrit ranked highest in stage L, followed by several prefracture mobility variables. Two considerations qualify this apparent divergence between the regression and machine-learning rankings: the mobility signal entered the models through 4 correlated encodings (the ordinal scale and 3 binary indicators), a constellation known to dilute the permutation importance attributed to each individual feature [23,24], and importance estimates, particularly in stage L, showed wide fold-to-fold variability. Prefracture functional status therefore remained a consistent contributor across both analytic approaches, whereas the prominence of individual laboratory markers should not be read as evidence of biological relevance.

Using the predefined decision policy, no HIGH-to-LOW misclassification occurred in the prespecified analysis. Under nested cross-validation, such errors occurred in 9 of 10 partitions and averaged 2.6. The decision policy therefore reduces extreme misclassification rather than eliminating it, and the reduction relative to the baseline was present in all 10 partitions. This profile reflects the predefined decision policy rather than the probabilistic models alone. The 2 directions differ in clinical consequence: LOW-to-HIGH errors overestimate mobility and may result in insufficient rehabilitation and support after discharge, whereas HIGH-to-LOW errors overestimate the need for support. Both were less frequent than with the baseline (5.7 vs 15.0 and 2.6 vs 7.5). Five of 158 (3.2%) LOW-to-HIGH errors were observed; all showed elevated *P*_high_ values substantially exceeding the threshold, with predicted HIGH probabilities higher than those of most correctly classified HIGH patients. This pattern suggests that these cases presented with clinical profiles resembling higher-mobility patients at admission and that the discrepancy between predicted and actual outcomes was driven by in-hospital processes not captured by admission variables.

The favorable extreme-error profile was achieved in part through conservative assignment to the intermediate category: among the 158 patients, MID was predicted in 102 (64.6%) patients, compared with an observed prevalence of 59 (37.3%), with correspondingly low MID precision (0.42; Figure 4). This behavior is an intended property of the decision policy, which defaults to MID whenever neither extreme probability exceeds its threshold. The higher MID recall relative to the baseline should accordingly be understood as trading overassignment to the middle category for protection against extreme errors, not as improved discrimination of the MID class per se. The same behavior limits detection of LOW mobility: LOW recall was 0.207 in the prespecified analysis and 0.117 under nested evaluation, compared with 0.407 for the baseline. The baseline reached this recall by assigning LOW liberally, at the cost of 7.5 HIGH-to-LOW errors vs 2.6 for the 2-stage framework.

### Comparison With Prior Work

Our cohort corresponds closely to contemporary descriptions of hospitalized patients with OVF—advanced age, female predominance, and substantial comorbidity burden align with established epidemiological data [1] and prior OVF inpatient series [3,6,25]. The distribution of fracture severity and operative treatment reflects recent OF classification-based series [26,27].

The finding that prefracture functional capacity rather than fracture grade or treatment predicts discharge mobility is consistent with prior OVF data [3,4] and broader vertebral fragility fracture cohorts demonstrating that functional recovery is largely driven by preexisting mobility. In contrast, OVF-specific data linking individual laboratory parameters to early functional recovery remain limited [25], though evidence from orthogeriatric fragility fracture cohorts indicates that anemia, systemic illness, and impaired metabolic reserve are associated with poorer short-term functional trajectories [28-31].

From a modeling perspective, the moderate discrimination observed (AUCs of 0.693 and 0.625 for the binary components) is consistent with comparable spine surgery prediction studies [32,33] and with systematic evidence showing that machine learning does not consistently outperform regression when dominant baseline predictors capture the principal prognostic signal [34]. Major misclassifications occurred exclusively in the LOW-to-HIGH direction in the prespecified analysis, whereas HIGH-to-LOW errors also occurred under nested evaluation; minor misclassifications were confined to adjacent mobility states, consistent with the recognized difficulty of separating neighboring functional categories in geriatric fracture populations and with evidence that in-hospital processes substantially influence short-term recovery [35-37]. Taken together, the added complexity was not justified by gains in discrimination or calibration, which were modest or absent; its value lay in 3-class differentiation and the reduction of extreme misclassifications. Because the baseline assigned classes by the maximum probability, this reduction is attributable to the combination of the stage-wise probabilities with the predefined decision policy rather than to the machine-learning components alone. Whether this trade-off warrants the additional complexity depends on the clinical weight assigned to avoiding extreme errors. The extreme-error profile depended on the stability of the stage L probabilities rather than on the thresholds: with the out-of-fold probabilities held fixed, no HIGH-to-LOW error occurred, whereas refitting the models for each partition produced such errors irrespective of whether the thresholds were reselected.

### Limitations

This was a single-center cohort from a certified level 1 spine center, which may limit generalizability. The local care pathway typically involved inpatient geriatric rehabilitation after operative treatment, whereas conservatively treated patients were more often discharged home. Together with the center’s referral and admission profile of predominantly older patients with osteoporotic thoracolumbar fractures requiring inpatient treatment, these characteristics may have shaped the observed distribution of discharge mobility. Transportability therefore depends on comparable case mixes and rehabilitation pathways, and external validation in other settings is required before clinical implementation. Because the decision-policy thresholds were derived from the same pooled out-of-fold predictions that underlie the reported performance estimates, these estimates should be regarded as best-case internal results. The nested reevaluation quantified this optimism. On identical partitions, nested and fixed-threshold evaluations differed by 0.002 in macro-*F*_1_, suggesting that the numerical threshold values were relatively stable across these partitions. Only the nested evaluation, however, provides a leakage-free estimate of the complete decision pipeline and is therefore the more realistic estimate of performance: macro-*F*_1_ was 0.388 (SD 0.031) across 10 partitions, and HIGH-to-LOW errors were no longer absent. Discrimination showed the same pattern, with an AUC of 0.663 for stage A and 0.594 for stage L across partitions. Structural hyperparameters were selected on this cohort and were not nested, which remains an unquantified source of optimism. Prediction was restricted to admission variables and did not account for dynamic in-hospital processes, including acute medical complications, delirium, and rehabilitation response, that may substantially influence short-term recovery. This restriction was a deliberate design choice to enable prediction at admission; incorporating the in-hospital course might improve predictive performance but would address a different clinical question, placing an inherent ceiling on the performance achievable with admission-only data. The 3-level outcome definition increases modeling complexity compared with binary end points and may contribute to moderate discrimination. Unmeasured factors such as pain burden, rehabilitation intensity, and social support were not captured. The stage A calibration slope below 1 indicates some overfitting of the predicted probabilities; the stage L slope was estimated too imprecisely to be informative (95% CI 0.156‐1.600, including 1), given the limited number of LOW outcomes (n=29). These calibration properties suggest that the model is better suited to risk stratification than to precise individual probability estimation; external validation and, if necessary, recalibration would be required before probability-based clinical use. The moderate sample size relative to the 31 candidate predictors may have contributed to this instability [38], consistent with the uncertainty seen in the calibration and feature-importance estimates.

Strengths include the prospective design, standardized outcome assessment, clinically well-characterized real-world cohort, and the use of an ordinal mobility end point reflecting clinically relevant gradations. Model evaluation included detailed misclassification analysis distinguishing major from adjacent errors, enabling structured assessment beyond global performance metrics.

### Conclusions

In hospitalized patients with osteoporotic thoracolumbar fractures, discharge mobility is primarily determined by prefracture functional reserve. The 2-stage framework achieved moderate multiclass discrimination and reduced extreme LOW-HIGH misclassification relative to a mobility-only baseline; this reduction persisted under nested cross-validation. Using routinely available variables at admission, the model may support early risk stratification, with potential applications in geriatric comanagement and physiotherapy resource planning. External validation in multicenter cohorts is a prerequisite before clinical implementation.

## Supplementary material

10.2196/100486 Multimedia Appendix 1 Nested evaluation of the decision policy: results by cross-validation partition.

## Abbreviations

AIC: Akaike information criterion
AUC: area under the curve
CCI: Charlson Comorbidity Index
CRP: C-reactive protein
eGFR: estimated glomerular filtration rate
OF: osteoporotic fracture
OVF: osteoporotic vertebral fracture
