# Supplementary material for: Discharge Mobility After Osteoporotic Thoracolumbar Fractures Using a 2-Stage Machine Learning Prediction Model: Prospective Cohort Study
Source: JMIR Form Res. 2026 Oct 29;10:e100486. doi: 10.2196/100486 (PMC13718535; doi:10.2196/100486)
Supplement: Supplementary file 1 [file formative-v10-e100486-s001.docx]

# **Multimedia Appendix 1. Nested evaluation of the decision policy: results by cross-validation partition**

Table A1. Performance by partition (n = 158 per partition; 29 LOW, 59 MID, 70 HIGH). Major errors are misclassifications between LOW and HIGH.

| **Partition** | **Analysis** | **Macro-F1** | **Recall LOW** | **Recall MID** | **Recall HIGH** | **HIGH-to-LOW** | **LOW-to-HIGH** | **Major errors** |
| --- | --- | --- | --- | --- | --- | --- | --- | --- |
| P1 | Nested thresholds | 0.411 | 0.172 | 0.661 | 0.414 | 1 | 3 | 4 |
| P1 | Fixed thresholds | 0.406 | 0.172 | 0.644 | 0.414 | 1 | 3 | 4 |
| P1 | Baseline | 0.342 | 0.379 | 0.017 | 0.871 | 6 | 15 | 21 |
| P2 | Nested thresholds | 0.359 | 0.103 | 0.644 | 0.371 | 2 | 4 | 6 |
| P2 | Fixed thresholds | 0.369 | 0.138 | 0.627 | 0.371 | 2 | 4 | 6 |
| P2 | Baseline | 0.359 | 0.414 | 0.034 | 0.871 | 8 | 15 | 23 |
| P3 | Nested thresholds | 0.338 | 0.138 | 0.576 | 0.329 | 3 | 6 | 9 |
| P3 | Fixed thresholds | 0.335 | 0.172 | 0.559 | 0.300 | 3 | 6 | 9 |
| P3 | Baseline | 0.358 | 0.414 | 0.034 | 0.871 | 9 | 15 | 24 |
| P4 | Nested thresholds | 0.388 | 0.069 | 0.695 | 0.457 | 2 | 4 | 6 |
| P4 | Fixed thresholds | 0.410 | 0.138 | 0.678 | 0.443 | 3 | 4 | 7 |
| P4 | Baseline | 0.350 | 0.414 | 0.017 | 0.871 | 6 | 15 | 21 |
| P5 | Nested thresholds | 0.440 | 0.103 | 0.814 | 0.471 | 0 | 6 | 6 |
| P5 | Fixed thresholds | 0.435 | 0.103 | 0.814 | 0.457 | 1 | 6 | 7 |
| P5 | Baseline | 0.339 | 0.379 | 0.017 | 0.871 | 8 | 15 | 23 |
| P6 | Nested thresholds | 0.365 | 0.069 | 0.695 | 0.400 | 1 | 8 | 9 |
| P6 | Fixed thresholds | 0.382 | 0.103 | 0.695 | 0.400 | 1 | 8 | 9 |
| P6 | Baseline | 0.359 | 0.414 | 0.034 | 0.871 | 8 | 15 | 23 |
| P7 | Nested thresholds | 0.385 | 0.103 | 0.610 | 0.471 | 5 | 8 | 13 |
| P7 | Fixed thresholds | 0.379 | 0.138 | 0.610 | 0.414 | 6 | 7 | 13 |
| P7 | Baseline | 0.347 | 0.414 | 0.017 | 0.871 | 8 | 15 | 23 |
| P8 | Nested thresholds | 0.370 | 0.103 | 0.610 | 0.429 | 7 | 8 | 15 |
| P8 | Fixed thresholds | 0.358 | 0.138 | 0.593 | 0.371 | 7 | 7 | 14 |
| P8 | Baseline | 0.335 | 0.414 | 0.000 | 0.871 | 8 | 15 | 23 |
| P9 | Nested thresholds | 0.413 | 0.138 | 0.729 | 0.414 | 1 | 6 | 7 |
| P9 | Fixed thresholds | 0.413 | 0.138 | 0.712 | 0.429 | 2 | 7 | 9 |
| P9 | Baseline | 0.355 | 0.448 | 0.017 | 0.871 | 8 | 15 | 23 |
| P10 | Nested thresholds | 0.414 | 0.172 | 0.661 | 0.429 | 4 | 4 | 8 |
| P10 | Fixed thresholds | 0.414 | 0.172 | 0.678 | 0.414 | 2 | 4 | 6 |
| P10 | Baseline | 0.353 | 0.379 | 0.034 | 0.871 | 6 | 15 | 21 |
| **Mean (SD)** | **Nested thresholds** | **0.388 (0.031)** | **0.117 (0.037)** | **0.669 (0.068)** | **0.419 (0.045)** | **2.6 (2.2)** | **5.7 (1.9)** | **8.3 (3.4)** |
| **Mean (SD)** | **Fixed thresholds** | **0.390 (0.031)** | **0.141 (0.025)** | **0.661 (0.072)** | **0.401 (0.045)** | **2.8 (2.1)** | **5.6 (1.7)** | **8.4 (3.1)** |
| **Mean (SD)** | **Baseline** | **0.350 (0.009)** | **0.407 (0.022)** | **0.022 (0.011)** | **0.871 (0.000)** | **7.5 (1.1)** | **15.0 (0.0)** | **22.5 (1.1)** |

Table A2. Thresholds selected within each outer fold of the nested analysis. Constraints met indicates whether at least one candidate configuration satisfied the predefined constraints (HIGH recall ≥ 0.40, LOW recall ≥ 0.18, predicted MID proportion ≤ 0.75); where no configuration did, the same ranking was applied without the constraints. Guard denotes the maximum p_high permitted for a LOW assignment.

| **Partition** | **Fold** | **n training** | **n held-out** | **p_high** | **p_high conditional** | **p_low** | **Guard** | **Constraints met** |
| --- | --- | --- | --- | --- | --- | --- | --- | --- |
| P1 | 1 | 126 | 32 | 0.61 | 0.54 | 0.46 | 0.15 | no |
| P1 | 2 | 126 | 32 | 0.66 | 0.52 | 0.5 | 0.15 | no |
| P1 | 3 | 126 | 32 | 0.63 | 0.52 | 0.5 | 0.15 | no |
| P1 | 4 | 127 | 31 | 0.63 | 0.6 | 0.5 | 0.15 | no |
| P1 | 5 | 127 | 31 | 0.63 | 0.54 | 0.48 | 0.15 | no |
| P2 | 1 | 126 | 32 | 0.66 | 0.56 | 0.44 | 0.15 | yes |
| P2 | 2 | 126 | 32 | 0.66 | 0.6 | 0.5 | 0.15 | no |
| P2 | 3 | 126 | 32 | 0.66 | 0.6 | 0.44 | 0.15 | no |
| P2 | 4 | 127 | 31 | 0.66 | 0.52 | 0.46 | 0.15 | no |
| P2 | 5 | 127 | 31 | 0.63 | 0.52 | 0.48 | 0.15 | yes |
| P3 | 1 | 126 | 32 | 0.66 | 0.52 | 0.5 | 0.15 | no |
| P3 | 2 | 126 | 32 | 0.66 | 0.52 | 0.46 | 0.15 | no |
| P3 | 3 | 126 | 32 | 0.61 | 0.6 | 0.48 | 0.15 | no |
| P3 | 4 | 127 | 31 | 0.61 | 0.6 | 0.46 | 0.15 | no |
| P3 | 5 | 127 | 31 | 0.66 | 0.52 | 0.5 | 0.15 | no |
| P4 | 1 | 126 | 32 | 0.66 | 0.52 | 0.5 | 0.15 | no |
| P4 | 2 | 126 | 32 | 0.66 | 0.52 | 0.5 | 0.15 | no |
| P4 | 3 | 126 | 32 | 0.61 | 0.52 | 0.48 | 0.15 | no |
| P4 | 4 | 127 | 31 | 0.66 | 0.58 | 0.46 | 0.15 | no |
| P4 | 5 | 127 | 31 | 0.61 | 0.56 | 0.5 | 0.15 | no |
| P5 | 1 | 126 | 32 | 0.66 | 0.56 | 0.5 | 0.15 | no |
| P5 | 2 | 126 | 32 | 0.63 | 0.52 | 0.5 | 0.15 | no |
| P5 | 3 | 126 | 32 | 0.66 | 0.54 | 0.5 | 0.15 | no |
| P5 | 4 | 127 | 31 | 0.63 | 0.6 | 0.5 | 0.15 | no |
| P5 | 5 | 127 | 31 | 0.66 | 0.52 | 0.5 | 0.15 | no |
| P6 | 1 | 126 | 32 | 0.63 | 0.6 | 0.5 | 0.15 | no |
| P6 | 2 | 126 | 32 | 0.61 | 0.6 | 0.48 | 0.15 | no |
| P6 | 3 | 126 | 32 | 0.63 | 0.52 | 0.48 | 0.15 | no |
| P6 | 4 | 127 | 31 | 0.63 | 0.54 | 0.5 | 0.15 | no |
| P6 | 5 | 127 | 31 | 0.61 | 0.56 | 0.44 | 0.15 | yes |
| P7 | 1 | 126 | 32 | 0.66 | 0.54 | 0.5 | 0.15 | no |
| P7 | 2 | 126 | 32 | 0.63 | 0.52 | 0.5 | 0.15 | no |
| P7 | 3 | 126 | 32 | 0.63 | 0.6 | 0.48 | 0.15 | no |
| P7 | 4 | 127 | 31 | 0.66 | 0.52 | 0.44 | 0.15 | no |
| P7 | 5 | 127 | 31 | 0.63 | 0.52 | 0.5 | 0.15 | no |
| P8 | 1 | 126 | 32 | 0.61 | 0.52 | 0.48 | 0.15 | no |
| P8 | 2 | 126 | 32 | 0.66 | 0.58 | 0.46 | 0.15 | no |
| P8 | 3 | 126 | 32 | 0.61 | 0.52 | 0.46 | 0.15 | yes |
| P8 | 4 | 127 | 31 | 0.63 | 0.52 | 0.5 | 0.15 | no |
| P8 | 5 | 127 | 31 | 0.66 | 0.52 | 0.46 | 0.15 | no |
| P9 | 1 | 126 | 32 | 0.66 | 0.54 | 0.44 | 0.15 | no |
| P9 | 2 | 126 | 32 | 0.66 | 0.52 | 0.46 | 0.15 | no |
| P9 | 3 | 126 | 32 | 0.66 | 0.56 | 0.5 | 0.15 | no |
| P9 | 4 | 127 | 31 | 0.61 | 0.54 | 0.46 | 0.15 | no |
| P9 | 5 | 127 | 31 | 0.61 | 0.58 | 0.5 | 0.15 | yes |
| P10 | 1 | 126 | 32 | 0.63 | 0.58 | 0.48 | 0.15 | no |
| P10 | 2 | 126 | 32 | 0.61 | 0.58 | 0.44 | 0.15 | no |
| P10 | 3 | 126 | 32 | 0.61 | 0.58 | 0.44 | 0.15 | no |
| P10 | 4 | 127 | 31 | 0.66 | 0.52 | 0.44 | 0.15 | yes |
| P10 | 5 | 127 | 31 | 0.66 | 0.52 | 0.5 | 0.15 | no |
| **Median (range)** |  |  |  | **0.63 (0.61–0.66)** | **0.54 (0.52–0.60)** | **0.48 (0.44–0.50)** | **0.15 (0.15–0.15)** | **6 of 50** |
